# A comparison of open or laparoscopic colectomy outcomes for the management of ischemic colitis using the ACS-NSQIP database

**DOI:** 10.1016/j.sipas.2023.100188

**Published:** 2023-06-03

**Authors:** Ben S. Duggan, Tim Becker, Genaro A. DeLeon, Varun Rao, Kevin Y. Pei

**Affiliations:** a Indiana University School of Medicine, 340 W 10th St, Indianapolis, IN 46202, United States; b Parkview Health Graduate Medical Education, 2200 Randallia Dr., Fort Wayne, IN 46805, United States

**Keywords:** Ischemic colitis, Colectomy, Laparoscopy, NSQIP, Colorectal

## Abstract

**Introduction:**

Ischemic colitis is a common manifestation of intestinal ischemia and is potentially a surgical emergency. Although such surgical emergencies were historically approached via open exploration, it is uncertain if there is a role for minimally invasive techniques. This study compares open vs laparoscopic colectomy techniques in the management of ischemic colitis.

**Methods:**

Using the American College of Surgeons (ACS) National Surgical Quality Improvement Program (NSQIP) database, patients with ischemic colitis undergoing colectomy from 2005 to 2019 were compared. The primary outcome of interest was 30-day mortality. Additional outcomes of interest were procedure related readmission, procedure related reoperation, length of stay, surgical site infections (SSI), septic shock, and other complications. Outcomes of interest were compared using multivariate logistic regression.

**Results:**

7,928 patients had ischemic colitis with 7,209 undergoing open colectomy and 719 undergoing laparoscopic colectomy. The mortality rate was significantly lower using a laparoscopic approach compared to open (6.4% vs 26%, *p*=<0.001) and associated with a lower odd of mortality (OR 0.58; 95% CI [0.35, 0.95]). Procedure related reoperation was lower in the laparoscopic group (6.5% vs 11%, *p*<0.001), but multivariate analysis was not significant (OR 0.65; 95% CI [0.43,1]). Readmission rates were not statistically different (12% vs 10%, *p* = 0.2). Postoperative length of stay (7 vs 12 days, *p*=<0.001), septic shock (6.7% vs 27%, *p*=<0.001), and organ space SSI (3.2% vs 6.9%, *p*=<0.001) were significantly decreased using a laparoscopic approach.

**Discussion:**

30-day postoperative mortality was significantly lower using a laparoscopic. Patients that had a laparoscopic colectomy had shorter hospital stays. While patients that underwent laparoscopic procedures tended to be less sick, multivariate analysis showed decreased rates of sepsis and surgical site infections compared to open colectomies when correcting for these factors.

**Conclusion:**

Laparoscopic colectomy may be a better surgical approach for patients with ischemic colitis compared to open colectomy.

## Highlights

• Ischemic colitis is potentially a surgical emergency associated with high rates of mortality and morbidity traditionally managed with open colectomies.
• The role for minimally invasive surgery in the management of ischemic colitis has not been fully established.
• Using the National Surgical Quality Improvement Database, a large national database, laparoscopic colectomies were associated with a lower a lower 30-day postoperative mortality compared to open colectomy.
• Minimally invasive techniques were also associated with decreased length of stay, sepsis, and surgical site infections.

## 1 Introduction

Ischemic colitis (IC) is a common manifestation of intestinal ischemia and is potentially a surgical emergency. The incidence of ischemic colitis is underreported but it accounts for approximately 0.1–1 in 1000 hospitalizations in the United States, representing the burden of the on the healthcare system [1], [2], [3]. Patient presentations vary from with mild self-resolving disease to an acute abdomen picture requiring emergency surgery which make the disease difficult to diagnose and manage [1,[3], [4], [5]]. This is increasingly evident with the current literature showing that at an estimated 14%–66% of IC patients require operative management and is estimated to be higher for patients with acute presentations [1,[3], [4], [5], [6]]. Although such surgical emergencies were historically approached via open exploration, it is uncertain if there is a role for minimally invasive techniques.

Due to the paucity of literature using multi-center databases or focusing on type of surgical intervention to manage ischemic colitis, we sought to compare open vs laparoscopic colectomy techniques in the management of ischemic colitis. We hypothesized that with the increased training and surgeon familiarity with laparoscopic techniques, laparoscopic colectomy (LC) would result in lower mortality than open colectomies (OC) for patients with ischemic colitis. Secondary outcomes of interest include length of stay, procedure related readmission, and procedure related reoperation.

## 2 Methods

Data was obtained from American College of Surgeons (ACS) National Surgical Quality Improvement Program (NSQIP) database from 2005 to 2019. The NSQIP database is a national database that includes detailed information about preoperative risk factors and 30-day surgical outcomes. Patients that underwent an open or laparoscopic colectomy for ischemic colitis were included in the analysis. The patients diagnosed with ischemic colitis were identified using ICD-9 codes 557.0, 557.1, 557.9 and ICD-10 codes K55.0, K55.1, K55.9. Open colectomies were identified using the CPT codes 44,140 – 44,160. Laparoscopic colectomies were identified using the CPT codes 44,188 and 44,204 – 44,212. The primary outcome of interest was 30-day mortality with secondary outcomes of interest being procedure related readmission, procedure related reoperation, length of stay, surgical site infections (SSI), septic shock, cardiovascular complications, respiratory complications, and renal compilations.

Statistical analysis was performed using R (v4.0.5) [7], dplyr (v1.0.9) [8], gt (v0.6.0) [9], and gtsummary (v1.6.0) [10]. Univariate analysis of the preoperative, operative, and postoperative variables was assessed between the open and laparoscopic cohorts using the Kruskal-Wallis or Wilcoxon signed-rank test for continuous variables and the Chi-squared test or Fisher's exact test for categorical variables, when appropriate. Significance was determined using a p-value less than 0.05. A multivariate logistic regression was performed for the outcomes of interest to control for differences in age, sex, race, BMI, ASA class, emergency cases, preoperative sepsis, and the other variable listed in Table 1.

**Table 1:** Univariate analysis of preoperative factors including demographics, disease statis, and labs for the laparoscopic and open colectomy approaches in patients with ischemic colitis.

Characteristic	Laparoscopic *N* = 719	Open *N* = 7209	P-value
Age	67 (56, 75)	69 (60, 78)	<0.001
Sex Female Male	475 (66%) 243 (34%)	4192 (58%) 3013 (42%)	<0.001
Race American Indian Or Alaska Native Asian Or Pacific Islander Black Or African American Native Hawaiian Or Pacific Islander White Unknown/Not Reported	4 (0.6%) 8 (1.1%) 44 (6.1%) 2 (0.3%) 607 (84%) 54 (7.5%)	36 (0.5%) 87 (1.2%) 762 (11%) 11 (0.2%) 5594 (78%) 719 (10.0%)	0.001
Hispanic Ethnicity	38 (6.1%)	294 (4.9%)	0.2
BMI	27 (23, 31)	27 (22, 32)	0.3
Smokes	156 (22%)	1928 (27%)	0.003
Diabetes mellitus No Non-Insulin Insulin	585 (81%) 71 (9.9%) 63 (8.8%)	5266 (73%) 878 (12%) 1065 (15%)	<0.001
Dyspnea No Moderate Exertion At Rest	642 (89%) 60 (8.3%) 17 (2.4%)	5796 (80%) 810 (11%) 603 (8.4%)	<0.001
Ventilator dependent	21 (2.9%)	1475 (20%)	<0.001
Open wound/wound infection	24 (3.3%)	524 (7.3%)	<0.001
Systemic Sepsis None Sepsis Septic Shock SIRS	526 (74%) 86 (12%) 29 (4.1%) 72 (10%)	2171 (31%) 1776 (25%) 1961 (28%) 1066 (15%)	<0.001
Superficial Incisional SSI	0 (0%)	17 (0.4%)	0.2
Deep Incisional SSI	0 (0%)	17 (0.4%)	0.2
Organ/Space SSI	4 (0.8%)	183 (4.1%)	<0.001
Pneumonia	6 (1.1%)	190 (4.3%)	<0.001
Blood transfusion	40 (5.6%)	683 (9.5%)	<0.001
Emergency	234 (33%)	5632 (78%)	<0.001
ASA classification 1-Healthy 2-Mild systemic disease 3-Severe systemic disease 4-Life threatening systemic disease 5-Moribund	11 (1.5%) 178 (25%) 368 (51%) 147 (20%) 15 (2.1%)	32 (0.4%) 444 (6.2%) 2342 (33%) 3578 (50%) 795 (11%)	<0.001
Days from Hospital Admission to Operation	0.0 (0.0, 3.0)	1.0 (0.0, 3.0)	0.001

## 3 Results

A total of 7928 patients were identified as having ischemic colitis in the database with 7209 undergoing open colectomies and 719 undergoing laparoscopic colectomies. There were 38 procedures where a laparoscopic and open approach were used. These cases were included in the OC cohort only. Table 1 shows the preoperative characteristics for the open and laparoscopic colectomy groups. The average age was 67 (95% CI: [56, 75]) and 69 (95% CI: [60, 78]) for the LC and OC groups (*p* <0.001), respectively. 66% of patients were female in the LC group compared to 58% in the OC group (*p* <0.001). Patients tended to be White (84% vs 78%) with patients identifying as Black or African American (6.1% vs 11%), Asian or Pacific Islander (1.1% vs 1.2%), American Indiana or Alaskan Native (0.6% vs 0.5%), and Native Hawaiian or Pacific Islander (0.3% vs 0.2%) less often (*p* = 0.001). The average BMI was the same at 27 (*p* = 0.3). 33% of the LC cases were labeled as an emergency compared to 78% of the OC groups (*p* < 0.001). Patients who underwent a laparoscopic colectomy tended to be less sick. Less of the laparoscopic colectomy patients had septic shock (4.7% vs 30%) or sepsis (12% vs 25%) and more LC patients had no signs of systemic sepsis (74% vs. 30%), (*p* < 0.001). LC patients had a normal white blood cell count compared to leukocytosis in OC patients (9 vs 13, *p* < 0.001). Rates of congestive heart failure (3.3% vs 7.5%), COPD (13% vs 20%), acute renal failure (2.6% vs 9.3%), and currently being on dialysis (4.5% vs 12%) were significantly higher for patients who underwent open colectomies (*p* <0.001).

Univariate analysis of the postoperative outcomes are shown in Table 2. 30-day mortality was significantly decreased in the laparoscopic colectomy cohort at 6.4% compared to 25% in the open colectomy group (*p* <0.001). When patients in both groups died, it tended to be 8 days after a laparoscopic procedure compared to 3 days after an open procedure (*p* = 0.019). Operations took longer in the LC group compared to the OC group (145 vs 113 min, *p* <0.001). Procedure related reoperations were lower for the laparoscopic cohort (6.5% vs 11%, *p* <0.001), but procedure related readmission was not statistically different (12% vs 10%, *p* = 0.2). Total hospital stay length was shorter for LC patients (7 days) compared to the OC patients (12 days) (*p* <0.001). Rates of superficial surgical site infections (SSI) (3.8% vs 5.3%, *p* = 0.079), deep incisional SSI (1.3% vs 1.8%, *p* = 0.3), organ space SSI (3.2% vs 6.9%, *p*<0.001), sepsis (6.7% vs 11%, *p* <0.001), and septic shock (6.7% vs 27%, *p*<0.001) were lower in the laparoscopic group compared to the open colectomy group. Postoperative rates of stroke (0.3% vs 1.7%, *p* = 0.003), deep vein thrombosis (DVT) (1.8% vs 3.6%, *p* = 0.013), myocardial infarction (0.3% vs 1.7%, *p* = 0.003), cardiac arrest (1.3% vs 5.2%, *p*<0.001), and acute renal failure (2.1% vs 6.6%, *p*<0.001) were also lower in the LC group compared to the OC group, respectively. When looking at all therapeutic vascular procedures (e.g. bypass or angioplasty) performed, LC patients had 2 procedures performed compared to 238 (3.3%) in the OC group (*p*=<0.001).

**Table 2:** Univariate analysis of postoperative outcomes comparing open and laparoscopic colectomy for patients with ischemic colitis.

Characteristic	Laparoscopic *N* = 719	Open *N* = 7209	P-value
Mortality	46 (6.4%)	1901 (26%)	<0.001
Days from Operation to Death	8 (3, 16)	5 (1, 13)	0.019
Length of total hospital stay	7 (4, 13)	12 (7, 21)	<0.001
Still in Hospital > 30 Days	7 (1.3%)	365 (8.2%)	<0.001
Total operation time	145 (103, 204)	113 (81, 156)	<0.001
Return to OR	58 (8.1%)	1240 (17%)	<0.001
Procedure Related Reoperation	34 (6.5%)	496 (11%)	<0.001
Procedure Related Readmission	63 (12%)	451 (10%)	0.2
Wound classification 1-Clean 2-Clean/Contaminated 3-Contaminated 4-Dirty/Infected	11 (1.5%) 437 (61%) 167 (23%) 104 (14%)	63 (0.9%) 1958 (27%) 2374 (33%) 2814 (39%)	<0.001
Superficial Surgical Site Infection	27 (3.8%)	380 (5.3%)	0.079
Deep Incisional SSI	9 (1.3%)	132 (1.8%)	0.3
Organ Space SSI	23 (3.2%)	494 (6.9%)	<0.001
Sepsis	48 (6.7%)	794 (11%)	<0.001
Septic Shock	48 (6.7%)	1932 (27%)	<0.001
Pneumonia	36 (5.0%)	1024 (14%)	<0.001
Ventilator 48 H After Surgery	61 (8.5%)	2495 (35%)	<0.001
Blood Transfusions	87 (12%)	2195 (30%)	<0.001
Diagnostic Vascular Procedure	0 (0%)	27 (0.4%)	0.2
Therapeutic Vascular Procedure	2 (0.3%)	238 (3.3%)	<0.001

The multivariate logistical regression results are shown in Table 3. Laparoscopic colectomies were associated with a decreased odds of mortality at 0.58 (95% CI [0.35, 0.95], *p* < 0.031). Procedure related reoperation and readmission had a lower odds ratio in the LC group at 0.65 (95% CI [0.43, 1], *p* = 0.052) and 0.95 (95% CI [0.68, 1.33], *p* = 0.75), respectively, but was not statistically significant. Additionally, our analysis found a laparoscopic approach resulted in a decreased chance in septic shock (0.43, 95% CI [0.22, 0.83], *p* = 0.012), transfusions (0.49, 95% CI [0.35, 0.68], *p* <0.001), pneumonia (0.44, 95% CI [0.24, 0.8], *p* = 0.007), and ventilator dependence 48 h after the case (0.41, 95% [0.26, 0.65], *p*<0.001), compared to open colectomies. Patients who underwent a laparoscopic colectomy had a lower odds of receiving a therapeutic vascular procedure at 0.08 (95% CI [0.01, 0.58], *p* = 0.013) compared to open colectomy patients. Logistic regression showed no statistical difference for stroke, myocardial infarction, cardiac arrest, DVT, or acute renal failure.

**Table 3:** Multivariate regression analysis of select postoperative outcomes following colectomy comparing laparoscopic vs. open approaches.

Complication	Estimated OR	95% CI	P-value
Mortality	0.58	(0.35, 0.95)	0.031
Procedure Related Reoperation	0.65	(0.43, 1)	0.052
Procedure Related Readmission	0.95	(0.68, 1.33)	0.76
Still in Hospital > 30 Days	0.25	(0.09, 0.7)	0.008
Return to OR	0.67	(0.45, 1)	0.05
Superficial surgical site infection	0.69	(0.41, 1.16)	0.161
Deep Incisional SSI	0.66	(0.24, 1.8)	0.418
Organ Space SSI	0.57	(0.33, 0.99)	0.047
Sepsis	0.52	(0.24, 1.1)	0.087
Septic Shock	0.43	(0.22, 0.83)	0.012
Blood Transfusions	0.49	(0.35, 0.68)	<0.001
Pneumonia	0.44	(0.24, 0.8)	0.007
Ventilator 48 H After Surgery	0.41	(0.26, 0.65)	<0.001
Therapeutic Vascular Procedure	0.08	(0.01, 0.58)	0.013

## 4 Discussion

Ischemic colitis has a large variety of clinical presentations, disease severity, and is associated with high mortality and morbidity [1,[3], [4], [5]]. Current literature has studied potential risk factors for mortality and morbidity; however, many of these studies have been limited to small, single-center patient populations focusing on patient comorbidities and surgical history. Using the ACS NSQIP database from 2005 to 2019, we identified many patients with ischemic colitis that underwent an open or laparoscopic colectomy. 9% of the cases in our study were laparoscopic. Ballian et al. and Patel et al. compared LC and OC for emergency procedures using the NSQIP database. They found 9.6% of cases from 2005 to 2008 and 11% from 2005 to 2018 [11,12] were performed laparoscopically, respectively. This demonstrates that surgeons are using laparoscopic approaches for emergency colectomies more. Patients who required surgery for ischemic colitis in our study tended to be older, with multiple comorbidities, worsening functioning status, and often required emergency surgery. In our study, patients in the laparoscopic cohort tended to be healthier, with lower ASA classifications, fewer comorbidities before, and were less often emergent cases. The ASA class was greater than three in 22.1% and 61% of laparoscopic and open colectomies, respectively. Previous studies had ASA greater than three in 16% and 30% of laparoscopic and open emergency colectomies [12]. Laparoscopic colectomies took longer than open colectomies (145 vs 113 min, *p* <0.001), which is shorter than previously reported [11,13,14].

Thirty-day mortality rates were the primary outcome of interest for our study. The NSQIP database only follows patients for 30 days after a procedure which precludes us from comparing long term mortality. Mortality rates for laparoscopic procedures were 6.4% compared to 25% for open cases. We corrected for preoperative risk factors and found that laparoscopic procedures had decreased odds of mortality OR 0.58 (95% CI [0.35, 0.95], *p* < 0.031). Mortality rates vary greatly in the literature but have previously been reported to be 0–60% [1,[3], [4], [5], [6]]. A 2017 study by Tseng et al. using the NSQIP database reported 25.3% as the 30-day postoperative mortality for patients with ischemic colitis who underwent colectomy using any approach. This is similar to our OC mortality rate, however, they did not comment on the effect of open or laparoscopic approaches [15]. Patel et al. found a similar decreased odds of mortality with emergency laparoscopic colectomies of 0.578 [12]. The rate of mortality with LC can likely be attributed to decreased risk of infection, pneumonia, ventilator requirements, decreased risk of other morbidities, and patient selection.

Patients who underwent a laparoscopic colectomy had significantly reduced morbidities compared to the open colectomy cohort. A laparoscopic approach was associated fewer reoperations. However, the multivariate analysis results were not statistically significant. Previously reoperation rates for IC are reported to be 20–46% [2,13,16], which is higher than what we found. Other studies looking at emergency LC and OC procedures showed a similar risk of reoperation [11,12]. Readmission was higher in the LC group (12% vs 10%), but not significant. Hospital stays were much shorter for the laparoscopic group (7 vs 12 days, *p* <0.001). Castleberry et al. reported longer hospital length of stay at 26 days +/- 22 days [2]. Many studies have shown that laparoscopic colectomies techniques reduce hospital stays [11,12]. Ischemic colitis has been shown to be associated with a high risk of infection and septic shock in several studies [11,12,14]. Patients undergoing an OC had significantly higher rates of SSI, sepsis, and septic shock. Laparoscopic approaches were associated with decreased odds of organ space SSI and septic shock. Previous rates of postoperative septic shock have been reported as 15% [2], 24% [13], 26.3% [15].

There were limitations to our study. First, the study is retrospective in nature and relied on data from the NSQIP database which can make it difficult to translate the results into clinical practice. Additionally, the NSQIP database only tracks patients for 30 days after their operation, but patients with IC have a poor long term mortality that we could not track [2]. Second, while the NSQIP database is a well coded, we are unable to ascertain the decision making that went in choosing between an open or laparoscopic approach. Third, we have no information about surgeon experience or how familiar they are with either technique, which likely impacts patient outcomes and which approach was used. Fourth, while the variables give an indication of how sick the patients were, given the wide range of presentations of ischemic colitis, it is difficult to assess the acuity of patient's presentations in each group.

## 5 Conclusion

Ischemic colitis is a common disease that often requires operative management. It is associated with high mortality and morbidity. Our study found that patients who underwent laparoscopic colectomies had decreased mortality and length of stay compared to patients that received an open colectomy. Further studies are needed to determine if our results are due to a true benefit of a laparoscopic approach. While there were several limitations to our study, this study shows that there is a place for laparoscopic colectomies in the management of patients with ischemic colitis and can be a safe alternative to an open approach.

## Funding sources

This research did not receive any specific grant from funding agencies in the public, commercial, or not-for-profit sectors.

## Data availability

The authors used the American College of Surgeons (ACS) National Surgical Quality Improvement Program (NSQIP) database for this study. The database is available to members of the ACS. However, the authors cannot redistribute the data used from the NSQIP database. Thus, no data is made available.

## Declaration of Competing Interest

The authors declare that they have no known competing financial interests or personal relationships that could have appeared to influence the work reported in this paper.
